# Supplementary figures and images for: Single-cell profiling of HDAC inhibitor-induced EBV lytic heterogeneity defines abortive and refractory states in B lymphoblasts
Source: PLoS Pathog. 2026 Mar 23;22(3):e1013610. doi: 10.1371/journal.ppat.1013610 (PMC13029708; doi:10.1371/journal.ppat.1013610)

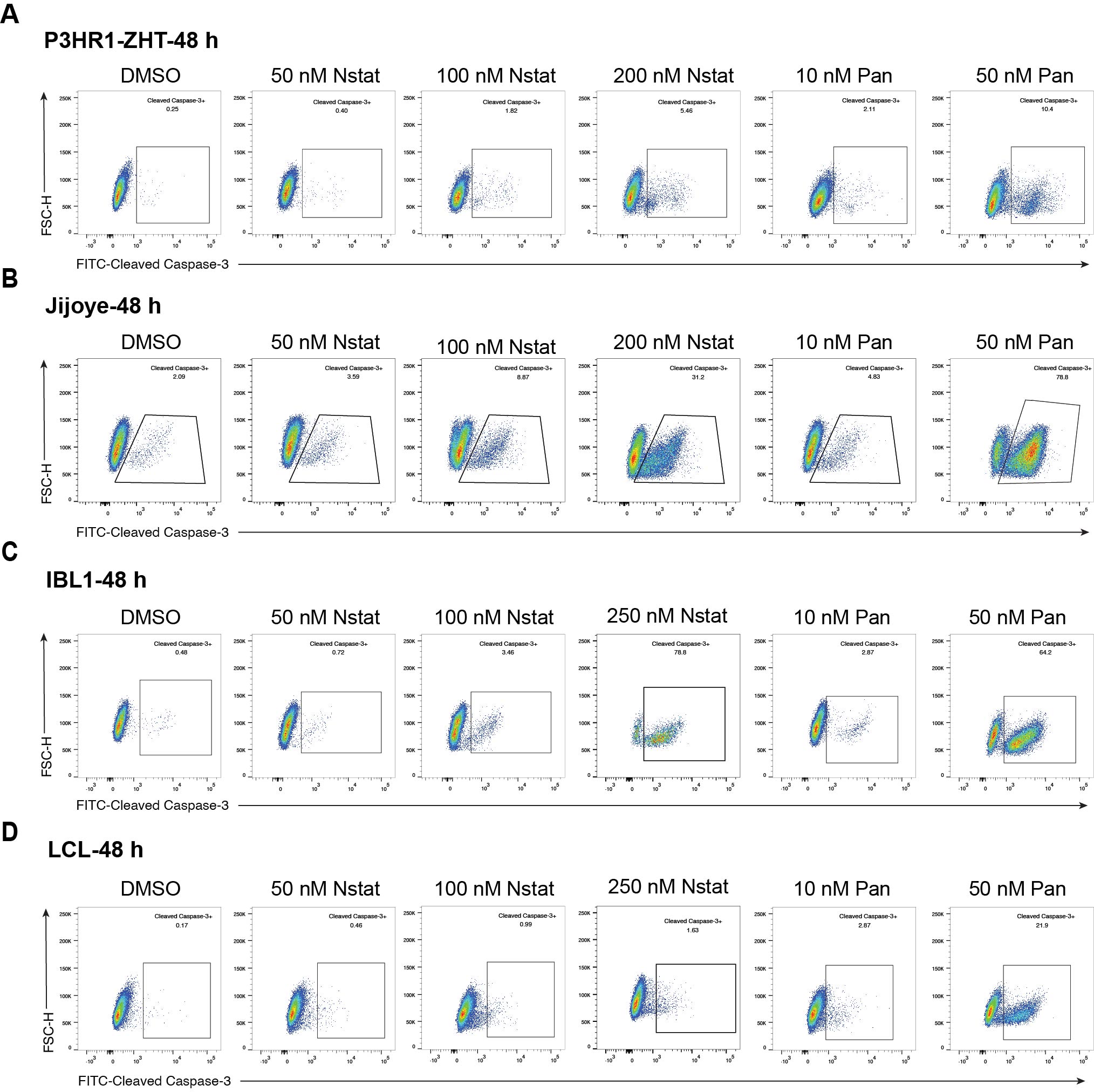

Supplement: S4 Fig — (A) Gating strategy for cleaved caspase-3 + P3HR1-ZHT cells treated with increasing concentration of HDAC inhibitor for 48h. (B) Gating strategy for cleaved caspase-3 + Jijoye cells treated with increasing concentration of HDAC inhibitor for 48h. (C) Gating strategy for cleaved caspase-3 + IBL1 cells treated with increasing concentration of HDAC inhibitor for 48h. (B) Gating strategy for cleaved caspase-3 + LCLs treated with increasing concentration of HDAC inhibitor for 48h. (JPG) [file ppat.1013610.s004.jpg]

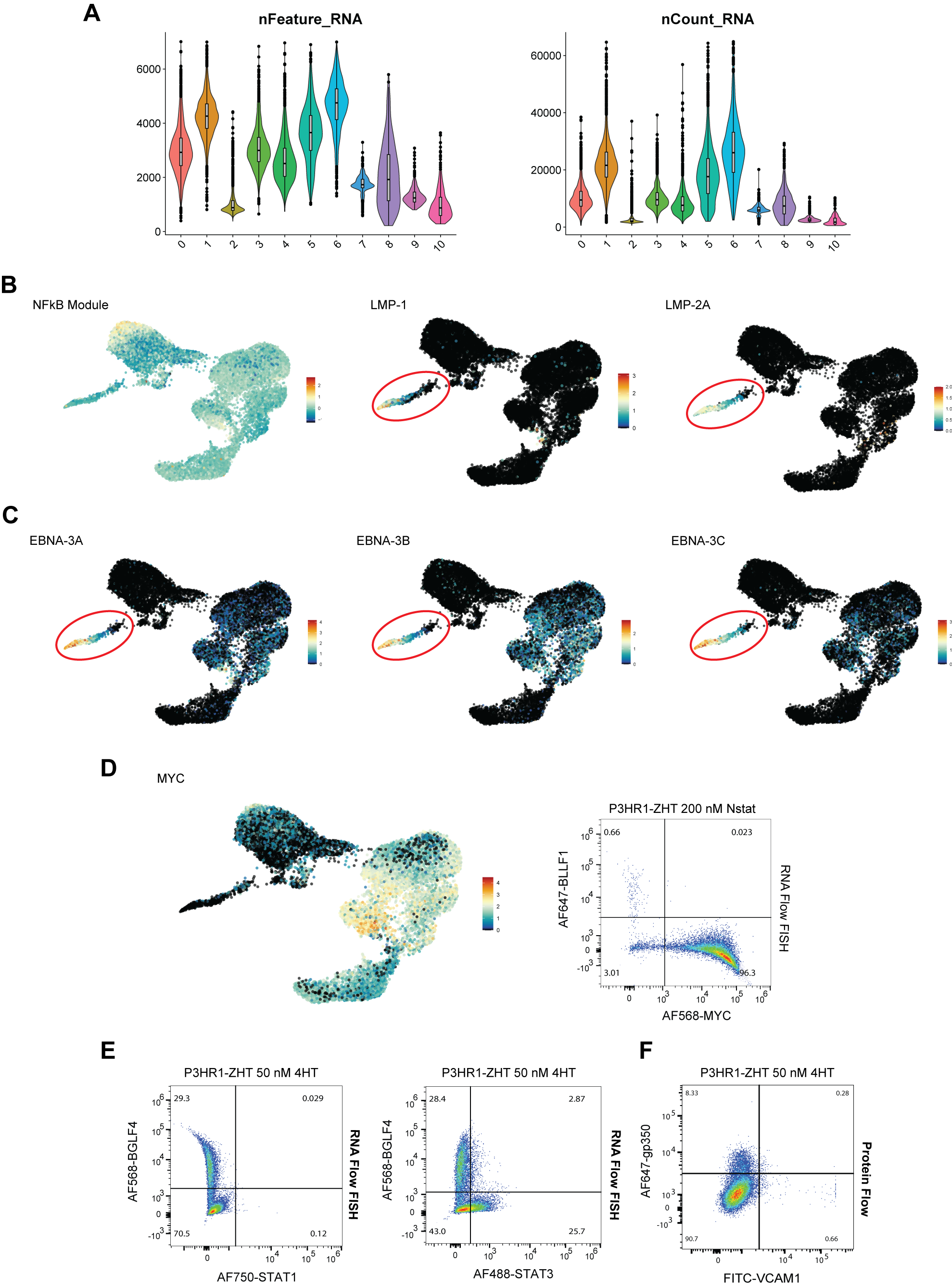

Supplement: S7 Fig — (A) QC RNA features by cluster. The number of unique RNAs expressed (genes, lncRNAs) per cell is depicted by nFeature_RNA. The total number of mapped reads per cell is depicted by nCount_RNA. (B) NFKB gene expression module: NFKB1, NFKBIA, NFKBIZ, NFKB2, BCL2A1 and UMAP expression of the viral genes LMP1 and LMP2A. Red circle denotes lytic cluster. (C) UMAP expression of the viral genes EBNA3A, EBNA3B, and EBNA3C. Red circle denotes lytic cluster. (D) UMAP expression of MYC and corresponding RNA Flow FISH validation probing for MYC and the late lytic gene BLLF1 following treatment with an HDAC inhibitor for 48h. (E) RNA Flow FISH plots of P3HR1-ZHT cells stimulated with 4HT for 24h and probed for expression of either STAT1 or STAT3 and the viral early lytic gene BGLF4. (F) Protein flow plot of P3HR1-ZHT cells treated with 4HT for 24h and stained for expression of VCAM1 and the late lytic protein gp350. (TIF) [file ppat.1013610.s007.tif]

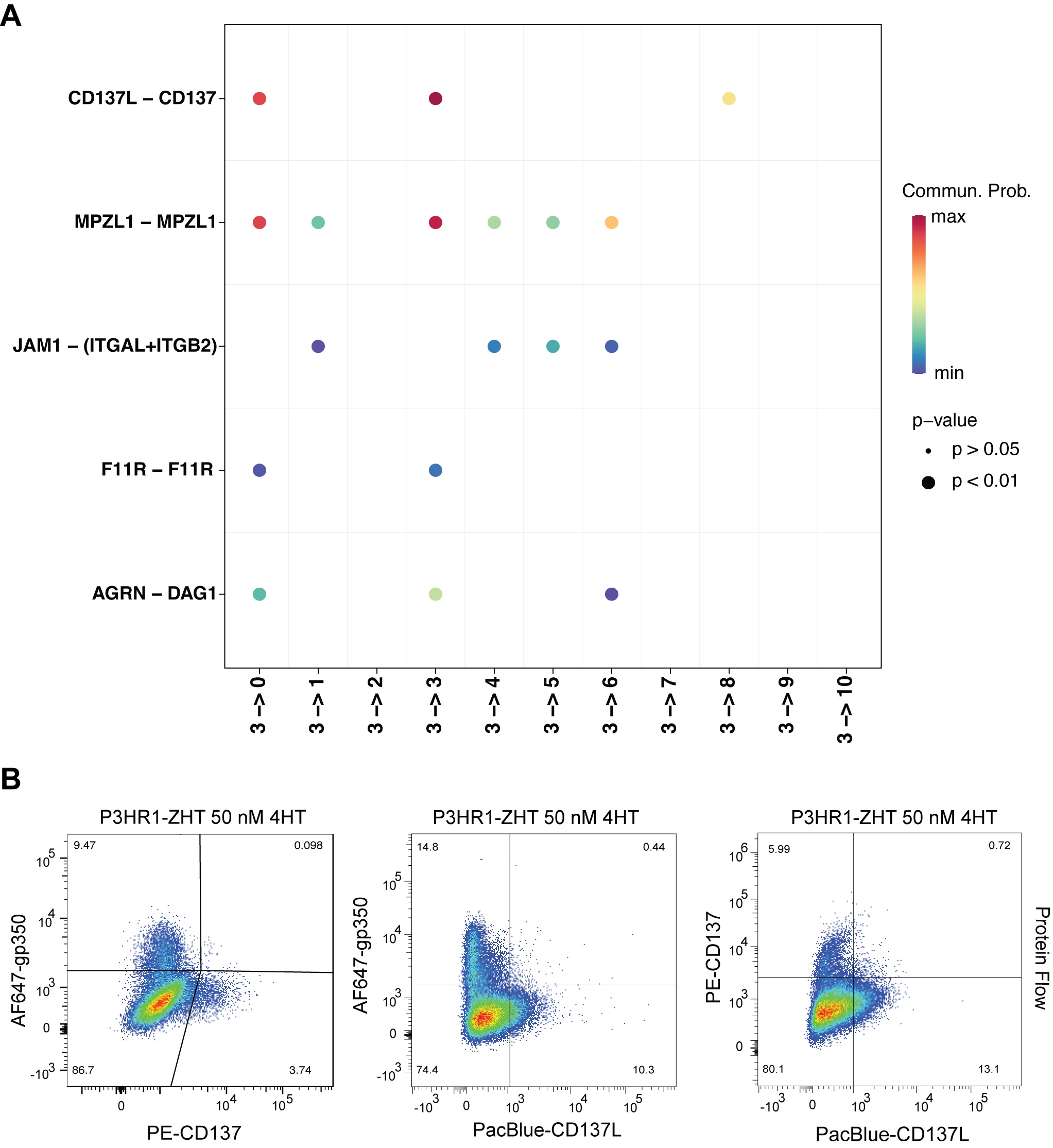

Supplement: S8 Fig — (A) Predicted communication probability of the top signaling pathways originating from cluster 3 (abortive cluster) from CellChat analysis. CD137L-CD137 signaling pathway was the only hit to show potential signaling between all three clusters of interest: refractory (0), abortive (3), and lytic (8). (B) Protein flow plots of P3HR1-ZHT cells treated with 4HT and stained for CD137, CD137L, and gp350. (TIF) [file ppat.1013610.s008.tif]

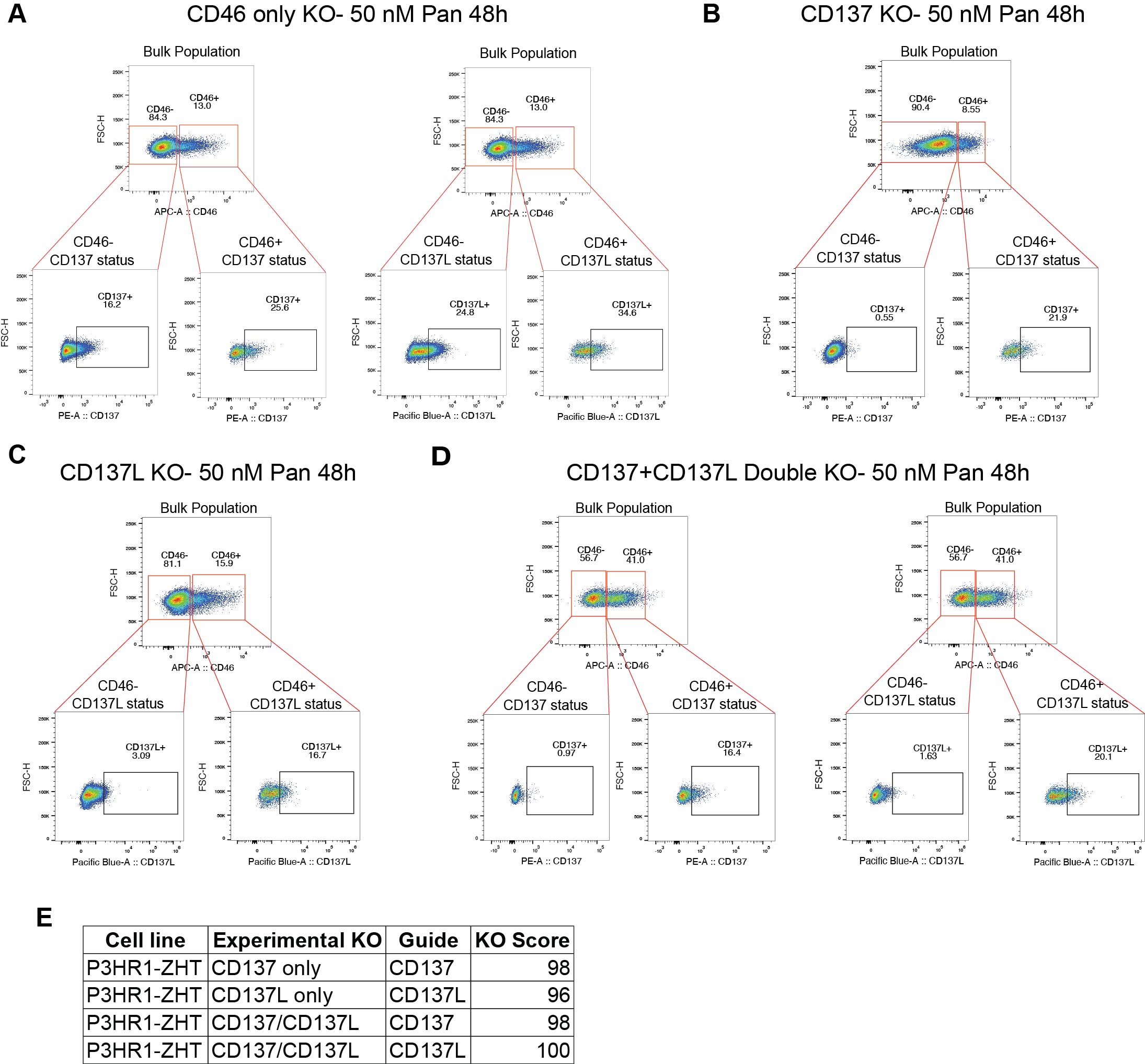

Supplement: S9 Fig — (A) CD46 was knocked out in P3HR1-ZHT cells. Cells were stimulated with 50 nM panobinostat for 48 h and stained for CD46, CD137, and CD137L. The CD46-positive and CD46-negative populations have comparable levels of CD137 and CD137L expressed. (B) CD46 and CD137 were knocked out in P3HR1-ZHT cells. Cells were stimulated with 50 nM panobinostat for 48 h and stained for CD46, and CD137. The CD46-negative population expressed significantly reduced CD137 following treatment. (C) CD46 and CD137L were knocked out in P3HR1-ZHT cells. Cells were stimulated with 50 nM panobinostat for 48 h and stained for CD46, and CD137L. The CD46-negative population expressed significantly reduced CD137L following treatment. (D) CD46, CD137, and CD137L were knocked out in P3HR1-ZHT cells. Cells were stimulated with 50 nM panobinostat for 48 h and stained for CD46, CD137, and CD137L. The CD46-negative population expressed significantly reduced CD137 and CD137L following treatment. (E) All experimental KO lines were sorted for the CD46-negative population to enrich for successfully transfected cells. All lines were sequenced and compared to the CD46 only KO. The Synthego ICE score tool was used to provide a KO score. (JPG) [file ppat.1013610.s009.jpg]
