# Supplementary material for: Single-cell profiling of HDAC inhibitor-induced EBV lytic heterogeneity defines abortive and refractory states in B lymphoblasts
Source: PLoS Pathog. 2026 Mar 23;22(3):e1013610. doi: 10.1371/journal.ppat.1013610 (PMC13029708; doi:10.1371/journal.ppat.1013610)
Supplement: Attachment — Submitted filename: HDACi_PlosPath_reviewer_responses_FINAL.docx [file ppat.1013610.s012.docx]

**Title:** Single-Cell Profiling of HDAC Inhibitor-Induced EBV Lytic Heterogeneity Defines Abortive and Refractory States in B Lymphoblasts

**ID:** PPATHOGENS-D-25-02508

**Author Responses to Reviewer Comments**

Original reviews in black

Author responses in blue

**Part I - Summary**
Please use this section to discuss strengths/weaknesses of study, novelty/significance, general execution and scholarship.

Reviewer #1: EBV causes human malignancies, and most EBV+ tumor cells contain latent EBV infection. "Lytic induction therapy" (converting EBV-infected tumor cells from the latent to lytic form of EBV infection) is being pursued as a potential method for treating latently-infected EBV+ tumors in humans, but a hurdle to using this therapy is the relatively low level of lytic infection induced by currently available drugs, including HDAC inhibitors. In this manuscript, the authors have used a variety of techniques (including single cell RNA-seq) to characterize the effects of a pan-HDAC inhibitor, panobinostat, versus a class I-specific HDAC inhibitor, nanatinostat, on the growth, survival, and lytic reactivation of four EBV-positive cell lines, including two related Burkitt lymphoma (BL) lines, (P3HR1-ZHT BL and Jijoye BL), one DLBCL line (IBL-1} and a newly infected lymphoblastoid cell line (LCL). The results of these experiments show that 1) the pan-HDAC inhibitor is more generally toxic compared to the HDAC I specific inhibitor; 2) the lytic induction efficiency of the HDAC inhibitors is much efficient in the two BL lines in comparison to the DLBCL and LCL lines; 3) even in the BL lines only a minority of the HDAC treated cells enter lytic infection, and often cells have only "abortive" lytic infection (expressing the BZLF1 immediate early gene but often not early lytic or late lytic genes); and 4) single-cell RNA-seq analysis of HDAC inhibitor treated P3HR1 BL cells identifies a "cluster 3" set of cells refractory to lytic induction that over-express a gene set consistent with NF-KB activation, including the CD137/CD137L genes. The authors suggest that the CD137/CD137L pathway may restrict lytic EBV reactivation in response to HDAC inhibitors. A strength of the manuscript is the use of several cutting edge techniques (including single cell RNA-seq and RNA hybridization probes) to identify how different EBV+ cells respond to HDAC inhibitors. Weaknesses are outlined below.

We thank the reviewer for their time and their positive consideration of the manuscript. We have addressed their comments in the sections below including additional experiments and analyses.

Reviewer #2: Epstein-Barr virus causes and maintains a latent reservoir in multiple malignancies making them difficult to treat. Viral reactivation using histone deacetylase inhibitors (HDACi) is being tested as a candidate therapeutic strategy yet has been met with limited success due to their inefficiency. In this manuscript, Haynes et al. explore potential mechanisms of why HDACis only lead to heterogenous responses. They phenotypically characterized the effects of class I HDACi nanatinostat and the pan-HDACi panabinostat on EBV+ lymphoblast cell lines (two Burkitt lymphoma: Jiyoye and P3HR1, one AIDS-associated immunoblastic lymphoma IBL1, and an LCL). HDACi treatment consistently led to an increase in cell cycle arrest yet had variable effects in reactivation. HDACi treatment induced lytic reactivation in BL cells but not in IBL1 and LCL. To gain insights on why majority of HDACi-treated cells are refractory to reactivation, they performed scRNAseq and compared it to their previous work. They show that abortive lytic cells have increased immune signaling genes such as STAT1/5A, CD137/L, and NFKB targets. Defining these genes that prevent lytic reactivation will inform future studies in developing more effective treatment for EBV cancers. Over-all, the study is descriptive in nature and relatively straightforward.

We thank the reviewer for their time and their positive consideration of the manuscript. We have addressed their comments below.

I only have a couple of minor comments:

1. For flow plots in Fig. 2 and 3, visualization of the BMRF1 subpopulation in the cell cycle plot and cleaved caspase subpopulation in the cell death-lytic panel are difficult to see. It would be better if they just express the subpopulations of the BMRF1 (Fig. 2) and active casp-3 (Fig. 3) as separate plots. It is unclear what the % BMRF1 is relative to in Fig. 2 --- is that from just the S phase? or total cells?

We have edited the BMRF1 labels in **Figure 2** to clarify that the BMRF1+ values are from the total cell population rather than a specific subset of cells. We have also included the BMRF1 subpopulation plots from Figure 2 as **Supplementary Figure 2** and the cleaved caspase-3 subpopulation plots from Figure 3 as **Supplementary Figure 4**.

2. Can you clarify why there are no BMRF1+Zombie violet+ double positives in Fig. 3? Don't late apoptotic cells eventually lose membrane integrity? Is it because the timepoint shown is too early to see this?

We have included Review Figure 1 to show that overall, there are limited double positive cells for BMRF1 and the cell death marker Zombie Violet. By including all cells in the downstream analysis, not just those within the lymphocyte gate (our lab’s naming scheme for healthy cells), we see a slight increase in the percentage of total dead cells (Zombie Violet+) (**Review Fig 1**). Some of these events are also likely debris or doublets which can present as false positives. This was the rationale for including a lymphocyte gate for downstream analysis. Additionally, Zombie Violet is a dye that is only able to stain cells with a permeable membrane. In this way we can visualize late apoptosis/cell death and early apoptosis with the cleaved caspase-3 marker simultaneously.

We have also included a snapshot below of **Supplementary Figure 4**. These plots show treated P3HR1-ZHT and Jijoye cells up to 72h post treatment. In the P3HR1-ZHT background, there is still limited double positive (BMRF1+, Zombie Violet+) cells by 72h. However, in the Jijoye line there is an increase in the percentage of double positive cells by 72h (top right quadrant). This data highlights the heterogeneous nature of responses between cell lines and shows that by 48h post treatment, it is too early to see the late apoptotic cells.

Review Figure 1: Limited double positive cells (BMRF1+ZombieViolet+) regardless of gating strategy**.** (*A*) Gating strategy for downstream analysis based on forward scatter (FSC) and side scatter (SSC). (*B*) Plots of treated P3HR1-ZHT cells that include cells only from the lymphocyte gate shown in A. Plots show the expression of BMRF1 as well as cell death and cleaved caspase-3. (*C*) Plots of treated P3HR1-ZHT cells that include all cells, including the lymphocyte gate (No FSC/SSC gating). These plots show the expression of BMRF1 as well as cell death and cleaved caspase-3 from all events collected.

Fig S4**:** P3HR1-ZHT cells were treated with 200 nM nanatinostat for 72h. Cells were collected every 24h and subsequently stained for BMRF1, cleaved caspase-3, and cell death. Jijoye cells were treated with 200 nM nanatinostat for 72h. Cells were collected every 24h and subsequently stained for BMRF1, cleaved caspase-3, and cell death.

3. Fig. 4. The HDACi-treated Jiyoye was compared to lytic induced P3HR1-ZHT cells. The differences in cellular background make it hard to draw robust conclusions from this comparison. Moreover, we also need to rule out that the more efficient induction of double positive cells in 4HT-treated P3HR1-ZHT cells is because ZTA is already expressed and just translocates to the nucleus (i.e. the barrier of breaking reactivation is inherently lower). A more straightforward comparison would be to examine lytic-induced Jiyoye (by Ig-crosslinking?) and HDACi-treated Jiyoye OR HDACi-treated P3HR1-ZHT with 4HT-treated or ig-crosslinked P3HR1-ZHT cells.

We thank the reviewer for this comment and have edited the paper for clarification. We agree that the barrier to successful lytic reactivation is inherently lower in the P3HR1-ZHT system treated with 4HT, and that this is the reason there are more double positive cells (BZLF1+, BLLF1+). We wished to highlight the fact that HDAC inhibition leads to a more heterogeneous and incomplete lytic reactivation compared to 4HT treatment in an inducible system. While the inducible system is useful for studying the biology of lytic reactivation, HDAC inhibitor treatment is more biologically relevant. We were interested in further investigating how lytic reactivation is successful vs unsuccessful following HDAC inhibition.

4. Fig. 5C. Lytic modules are not obvious. Having a zoomed inset of cluster 8 would help visualize the IE, E and L modules

We thank the reviewer for this comment and have edited Figure 5 to include a total lytic module UMAP that shows the expression of IE, E, and L genes (**Fig 5C**). We also included UMAP insets of just the lytic cluster (cluster 8) separated based on expression of IE, E, or L gene expression (**Fig 5D**). In this way, we hope to better show the progression of the lytic cycle within the lytic cluster of our single cell data.

**Part II – Major Issues: Key Experiments Required for Acceptance**
Please use this section to detail the key new experiments or modifications of existing experiments that should be absolutely required to validate study conclusions.

Generally, there should be no more than 3 such required experiments or major modifications for a "Major Revision" recommendation. If more than 3 experiments are necessary to validate the study conclusions, then you are encouraged to recommend "Reject".

Reviewer #1: 1. Although the authors suggest that the CD137/CD137L signaling pathway may be an important restriction factor preventing efficient lytic EBV reactivation in B cells, they do not include the essential experiments to support this statement. For example, does knock-down of either CD137 or CD137L increase the amount of lytic induction in response to HDAC inhibitors? Do the refractory LCL and DLBCL lines express higher levels of CD137/CD137L compared to the BL lines?

We thank the reviewer for this comment and agree it is a necessary question to address. To answer this question, we performed knockouts of the genes CD137, CD137L, and a double knockout of CD137+CD137L in the P3HR1-ZHT background using a Cas9-RNP approach. We combined our guides of interest with a guide targeting CD46, a non-essential surface protein, that we can use as a proxy for successful knockout (**Fig 8A**). We have included this data in main **Fig 8** and proof of concept data in **Supplementary Figure 9** (both shown below). Knockout of the receptor CD137 led to a significant increase in the percentage of cells that went lytic, at both early (BMRF1) and late (gp350) stages of the lytic cycle (**Fig 8B, C**). Interestingly, we observed no change in the percentage of cells that went lytic when the ligand CD137L was knocked out suggesting a possible alternative ligand or constitutive signaling by CD137 (**Fig 8B, C**). The double knockout (CD137+CD137L) phenocopies the receptor only knockout at both early and late stages of the lytic cycle (**Fig 8B, C**). The knockout of CD137 led to a ~70% increase in BMRF1 positive cells and a ~60% increase in gp350+ cells (**Fig 8B, C**). However, while the knockout of CD137 does lead to a significant increase in lytic cells, we still observe a disparity in the percentage of cells that progress from early to late lytic. Additionally, late lytic cells encompass ~2.5% of the total stimulated population in the CD137 knockout (**Fig 8C**). We conclude that CD137 receptor signaling does play a role in EBV lytic reactivation, but that there are other signaling pathways that play either an additive or redundant role in preventing successful lytic reactivation.

Revised Manuscript Fig 8: Knockout of CD137 leads to an increase in lytic protein expression at both the early and late stage of reactivation. (A) Workflow of knockout approach. P3HR1-ZHT cells were transfected with Cas9 and sgRNAs targeting CD46 and CD137, CD137L, or CD137+CD137L. CD46 is a non-essential surface protein that is used as a proxy for successful knockout. Cells recover for 72h following transfection. Cells are then stained for CD46 and sorted on the CD46-negative population, enriching for knockout cells. (B) P3HR1-ZHT knockout cells were stimulated with 50 nM panobinostat for 48h and stained for the early lytic protein BMRF1. One-way ANOVA was performed comparing values to the CD46 only knockout. (C) P3HR1-ZHT knockout cells were stimulated with 50 nM panobinostat for 48h and stained for the late lytic protein gp350. One-way ANOVA was performed comparing values to the CD46 only knockout. For panels B, C, n=3 **** p<0.0001.

Fig S9. Validation of knockouts. (*A*) CD46 was knocked out in P3HR1-ZHT cells. Cells were stimulated with 50 nM panobinostat for 48 h and stained for CD46, CD137, and CD137L. The CD46-positive and CD46-negative populations have comparable levels of CD137 and CD137L expressed. (*B*) CD46 and CD137 were knocked out in P3HR1-ZHT cells. Cells were stimulated with 50 nM panobinostat for 48 h and stained for CD46, and CD137. The CD46-negative population expressed significantly reduced CD137 following treatment. (*C*) CD46 and CD137L were knocked out in P3HR1-ZHT cells. Cells were stimulated with 50 nM panobinostat for 48 h and stained for CD46, and CD137L. The CD46-negative population expressed significantly reduced CD137L following treatment. (*D*) CD46, CD137, and CD137L were knocked out in P3HR1-ZHT cells. Cells were stimulated with 50 nM panobinostat for 48 h and stained for CD46, CD137, and CD137L. The CD46-negative population expressed significantly reduced CD137 and CD137L following treatment. (*E*) All experimental KO lines were sorted for the CD46-negative population to enrich for successfully transfected cells. All lines were sequenced and compared to the CD46 only KO. The Synthego ICE score tool was used to provide a KO score.

To address the second question, we treated the BL line Jijoye and the more refractory LCL and IBL1 lines with the HDAC inhibitor panobinostat and stained for either CD137 or CD137L. The BL line Jijoye expressed CD137 following treatment with 50 nM panobinostat (**Review Figure 2A, B**). IBL1 had a slight increase in the expression of CD137 following treatment that was dose dependent (**Review Figure 2A, B**). The LCL expressed some CD137 basally, but we observed an increase in the percentage of cells that expressed this marker following treatment with 10 nM panobinostat (**Review Figure 2A, B**). Overall, these cell lines express less CD137 following HDAC inhibition than the P3HR1 background. When we stained for CD137L, we observed limited expression of this marker in the Jijoye line even after HDAC inhibitor treatment (**Review Figure 2C, D**). However, CD137L expression was highest following 50 nM panobinostat treatment in both the IBL1 and LCL compared to the 10 nM panobinostat treatment (**Review Figure 2C, D**). These cell lines showed a general increase in the expression of CD137/CD137L following treatment with an HDAC inhibitor. However, we observed the highest overall expression of these markers in the P3HR1 background. These results suggest that while CD137/CD137L signaling may be involved across cell backgrounds following HDAC inhibition, this response is more prominent in the P3HR1 BL background. Based on our knockout data (shown above), it is also likely that CD137 receptor signaling is more important for lytic reactivation than expression of CD137L.

Review Figure 2: Responsive and refractory EBV-positive cell lines express varying levels of CD137/CD137L following HDAC inhibition. (*A*) Jijoye, IBL1, and LCLs were treated with the HDAC inhibitor panobinostat for 48 h and stained for the surface marker CD137. (*B*) Replicates of cell lines treated with HDAC inhibitor and stained for CD137 48 h post treatment. (*C*) Jijoye, IBL1, and LCLs were treated with the HDAC inhibitor panobinostat for 48 h and stained for the surface marker CD137L. (*D*) Replicates of cell lines treated with HDAC inhibitor and stained for CD137L 48 h post treatment.

2. Almost no information is provided regarding the effects of the HDAC inhibitors on the expression of EBV latency genes in the four different cell lines. The authors must have this information at the single-cell RNA-seq level in the P3HR1 cells, although they only show results for the LMP1 latency gene. In particular they need to show whether the HDAC inhibitors increase expression of the EBV LMP2A protein in the various cell lines (preferably at the protein level), given that LMP2A is known to decrease lytic EBV infection.

At the RNA level in the P3HR1 background, EBNA1 and the EBNA3s are expressed basally in the untreated samples (**Review Figure 3A, B,** blue circles). However, the overall expression of these viral latency genes is higher in the treated and successfully lytic population (**Review Figure 3A, B**, red circles). Due to the latency state of the P3HR1 line, LMP2A and LMP1 are not expressed in the resting untreated cells (**Review Figure 3C**, blue circles). However, both LMP1 and LMP2A are detected in the successful lytic population (**Review Figure 3C**, red circles). While HDAC inhibition does lead to increased expression of latency genes, including LMP2A, we only observe this in the successful lytic cells and not in our abortive lytic cluster.

Review Figure 3**.** Latency gene expression in HDAC inhibitor treated P3HR1-ZHT cells (*A*) UMAPs of EBNA1 and EBNA3A expression from untreated and HDAC inhibitor treated P3HR1-ZHT cells. (*B*) UMAPs of EBNA3B and EBNA3C expression from untreated and HDAC inhibitor treated P3HR1-ZHT cells. (*C*) UMAPs of LMP1 and LMP2A expression from untreated and HDAC inhibitor treated P3HR1-ZHT cells. In all UMAPs, the red circle highlights the lytic cluster while the blue circle highlights untreated, resting cells.

We also examined the expression of LMP2A at the protein level across all cell lines. We used cells treated with nanatinostat to maintain protein integrity since panobinostat is overall more toxic. While there is limited expression of LMP2A at the protein level in P3HR1 cells, we see expression of LMP2A increase in both Jijoye cells and the LCL following treatment (**Review Fig 4A**). The Jijoye line is responsive to HDAC inhibition while the LCL is a non-responsive line. Regardless of their responsiveness, LMP2A expression increases following treatment. We also examined LMP2A expression at the RNA level and performed qPCR across all cell lines treated with nanatinostat or panobinostat. We observed increased expression of LMP2A across all cell lines following treatment with an HDAC inhibitor regardless of whether the line was responsive or non-responsive (**Review Figure 4B**). We also examined the expression of LMP1 at the RNA level following treatment. We observed a similar pattern of increased latent gene expression following treatment regardless of responsiveness (**Review Figure 4C**). HDAC inhibition is known to lead to acetylation of the LMP2A promoter which is likely why we see a general increase in expression of this gene following treatment with either nanatinostat or panobinostat^1^. Since we observe an increase in LMP2A expression (at the gene and protein level) in both responsive and non-responsive lines and because we observe LMP2A expression exclusively in the successful lytic cells in our single cell dataset, we conclude that LMP2A (or LMP1) expression is not the main culprit behind this refractory/abortive phenotype.

Review Figure 4. Expression of LMP2A and LMP1 increases following HDAC inhibition regardless of responsiveness (*A*) Western blot probing for the EBV latency protein LMP2A. Cell lysates were collected from all cell lines treated with DMSO or 200 nM nanatinostat for 48 h. BJAB was used as a negative control. (*B*) qPCR was performed to measure the expression of LMP2A at the RNA level. Relative expression is shown as a percentage of a resting LCL. P3HR1-ZHT and Jijoye cells were treated with 50 nM panobinostat or 200 nM nanatinostat for 48 h. IBL1 and the LCL were treated with 10 nM panobinostat and 50 nM nanatinostat for 48 h to preserve sample integrity and avoid major cell death. (*C*) qPCR was performed to measure the expression of LMP1 at the RNA level. Relative expression is shown as a percentage of a resting LCL. P3HR1-ZHT and Jijoye cells were treated with 50 nM panobinostat and 200 nM nanatinostat for 48 h. IBL1 and the LCL were treated with 10 nM panobinostat and 50 nM nanatinostat for 48 h to preserve sample integrity and avoid major cell death.

3. As the lytic refractory "cluster 3" found by single cell RNA-seq in P3HR1 cells has an RNA expression pattern consistent with NF-KB (and/or LMP1) activation, and several papers have previously reported that NF-KB signaling (and LMP1) inhibits lytic EBV reactivation in EBV-infected B cells, the authors should compare the level of HDAC inhibitor induced canonical and non-canonical NF-KB proteins in the four different cell lines. Do the refractory lines have increased NF-KB activity in response to HDAC inhibitors?

We thank the reviewer for this comment and agree that NF-κB signaling is important in preventing lytic reactivation. In both this study and our previous work, we observed high NF-κB activity most closely associated with the abortive lytic clusters (cluster 3 in this study). Additionally, we observed that this activity was independent of LMP1 expression following both 4HT and HDAC inhibitor treatment. This led us to hypothesize that upstream pathways, likely immune signaling related, were driving this activity in abortive lytic cells. To address the question of whether canonical or non-canonical signaling may be more upregulated following HDAC inhibitor treatment, we tested all four cell lines for expression of RelA and RelB pre- and post-treatment.

All the cell lines used in this study express both RelA and RelB which are associated with canonical and non-canonical NF-κB signaling respectively. These proteins are also involved in cell survival and proliferation, hallmarks of cancer cell lines. We observed a general decrease in the expression of both RelA and RelB following treatment with either HDAC inhibitor (**Review Figure 5**). However, in the IBL1 line, there was not a significant change in protein expression in the untreated condition compared to nanatinostat treatment (**Review Figure 5**). Overall, the refractory lines did not have significantly more RelA or RelB protein expression following treatment than the responsive lines. Given this is a bulk western blot, we are unable to fully parse out how this protein expression is affected by cell death or whether it is only expressed in the abortive/refractory cells.

Review Figure 5. Canonical and non-canonical NF-kB family members are expressed following HDAC inhibition. Western blot for the proteins RelA and RelB. All cell lines were treated with DMSO, panobinostat, or nanatinostat for 48h.

Reviewer #2: None

**Part III – Minor Issues: Editorial and Data Presentation Modifications**
Please use this section for editorial suggestions as well as relatively minor modifications of existing data that would enhance clarity.

Reviewer #1: 1. The authors should mention that the P3HR1 "subclone" of Jijoye cells has an EBNA2 deletion and has "Wp-restricted" viral latency while the Jijoye BL cells have type III EBV latency. Thus neither cell line is a particularly good model system for studying lytic induction in human BL tumors, which generally have type I viral latency.

We thank the reviewer for this comment, and we have updated the text to reflect the relationship between P3HR1 and Jijoye, as well as the latency state of each. We have also cited our recent work from Willard et al, that shows recently isolated endemic Burkitt lymphoma lines exhibit various latency patterns which are distinct from the traditional latency I gene expression pattern. We recognize that the two BL lines we use in this study are not the traditional type I latency, but we hope this study highlights that different malignancies respond uniquely to HDAC inhibition regardless of latency status.

2. The authors should compare the cellular toxicity of the two different HDAC inhibitor drugs in EBV-infected Burkitt cells versus the same cells that have spontaneously lost the EBV genome (either EBV+ versus EBV-negative Akata BL cells or EBV+ versus EBV-negative Mutu BL cells) to determine if any of the drug induced cell killing is related to the presence of EBV infection.

We thank the reviewer for this comment and have included CC_50_ graphs of the EBV-negative Burkitt lymphoma line BL41 and the matched EBV infected BL41-B958 line treated with panobinostat and nanatinostat for 48 h (**Fig 1E, F**). CC_50_ concentrations were comparable between cell lines, highlighting that HDAC inhibition is toxic regardless of EBV status.

Reviewer #2: See above

Sources

1. Gerle B, Koroknai A, Fejer G, Bakos A, Banati F, Szenthe K, Wolf H, Niller HH, Minarovits J, Salamon D 2007. Acetylated Histone H3 and H4 Mark the Upregulated LMP2A Promoter of Epstein-Barr Virus in Lymphoid Cells. J Virol 81:. <https://doi.org/10.1128/jvi.01396-07>
